# Op Den Graeff

**Published:** 1875-10

**Authors:** 


### OP DEN GRAEFF.

A writer in the *Penn Monthly*, for September, gives an interesting account of the brothers op den Graeff who so largely figure in the early history of Pennsylvania. For the benefit of the very few families of the name (now written Up de Graff) residing in Pennsylvania, New York, Ohio and Indiana, we condense, from the source named, as follows:

"With Penn came a company of emigrants worthy of a more glorious immortality. These instead of waging war against all who differed from them, either in race or faith, desired to establish on these shores "peace and good will toward men," who dealt justly with negro and indian, and established and maintained perfect toleration toward every difference of opinion. Among these emigrants the history of none is more interesting or less known than of the German and Dutch. Of these some had undoubtedly embraced the doctrines of the Quakers from hearing them preached by Penn during his travels in Holland and Germany, while a large majority were Mennonites, who fled from persecution in Switzerland and on the Rhine. More than ever is the history of these interesting now, when American slavery has passed into history, since from four German Friends came the first protest, addressed to the Quarterly Meeting at Burlington, ever made against the holding of negroes as slaves. Their names were Garret Henderich, Dirck op den Graeff, Francis Daniel Pastorius and Abraham op den Graeff. Francis Daniel Pastorius is the hero of Whittier's Poem." Of the brothers op den Graeff the narrative continues: "They were probably the sons of Hermann op den Graeff, a Hennonite of Crefelt, in Rhinish Persia. The name is Dutch. They came over with Pastorius and a company, among whom were men whose descendants bearing Anglicized names, are scattered over Pennsylvania, New Jersey and Delaware. Reinert, Tisen (Tyson), Wilhelm, Strepers (Streeper), Peter Keurlis (Corlies or Corliss), Jan Simens (Simmons), Jan Lucken (Luckens), were some of these. They arrived in Philadelphia on the 6th of October, 1683, and on the 24th began their settlement at Germantown. Here they engaged in the manufacture of linen, particularly distinguishing themselves for the fineness of the fabric which they made. The charter for the incorporating of Germantown went into effect in 1691. Dirck was bailiff in 1693 and 1694, and Abraham a burgess in 1672. Their strongest claim to the grateful remembrance of posterity is the protest against slavery before mentioned, which was probably written by Pastorius. A short time after this dissensions upon doctrinal points began to arise among Friends, even in this then primitive country. George Keith, who afterward became one of the most effective ministers among the Episcopalians in America, differed with his Quaker brethren. The contest waxed hot, and, as is usual in such cases, spread from Chuch to State, Keith being accused of disrespect towards Dirck op den Graeff, in his capacity as magistrate. After several blasts and counterblasts, the matter ended in the disowning of Keith by the friends, and in Abraham op den Graeff's dissolving his connection wlth the society. Ecclesiastically it led to the foundation of the Episcopal Church in Pennsylvania; politically it was one of the principal reasons assigned for depriving Penn of the control of his province. After this, life flowed on smoothly enough with the op den Graeffs, ruffled only by the occasional troubles on account of their neglect of fences and the use of uncomplimentary adjectives. They were evidently a sturdy set, who respected no man merely because he was 'clothed with a little brief authority.' Their descendants, bearing the name of Updegraff, are few in number."

